# ADaM: augmenting existing approximate fast matching algorithms with efficient and exact range queries

**DOI:** 10.1186/1471-2105-15-S7-S1

**Published:** 2014-05-28

**Authors:** Nathan L Clement, Lee P Thompson, Daniel P Miranker

**Affiliations:** 1 Department of Computer Science, University of Texas at Austin, 2317 Speedway, Stop D9500, 78712 Austin, TX, USA

## Abstract

**Background:**

Drug discovery, disease detection, and personalized medicine are fast-growing areas of genomic research. With the advancement of next-generation sequencing techniques, researchers can obtain an abundance of data for many different biological assays in a short period of time. When this data is error-free, the result is a high-quality base-pair resolution picture of the genome. However, when the data is lossy the heuristic algorithms currently used when aligning next-generation sequences causes the corresponding accuracy to drop.

**Results:**

This paper describes a program, ADaM (**A**PF **D**NA **M**apper) which significantly increases final alignment accuracy. ADaM works by first using an existing program to align "easy" sequences, and then using an algorithm with accuracy guarantees (the APF) to align the remaining sequences. The final result is a technique that increases the mapping accuracy from only 60% to over 90% for harder-to-align sequences.

## Background

Recent advances in genomic sequencing technologies have caused a substantial increase in the rate of data produced, so much so that the problem of data procurement has shifted to a problem of data processing [1]. However, the corresponding error rate of these technologies has not altogether decreased, and in some cases has actually increased [2]. If these errors are not handled correctly, they can propagate through to later steps in the processing pipeline, lessening the significance of the biological conclusions.

Referenced genome assembly is one of the main techniques used in many next-generation sequencing applications. It works by assigning *reads* (genomic strings of varying length produced by sequence machines)to the best matching location on an existing reference genome through a process called *read mapping*. If a given read aligns with high confidence to a single location in the genome with all but one or two nucleotides, it can be said with high confidence that the difference is either due to sequencing error or a mutation in the genome of interest. If many reads have the same "error" in the same genomic position, the possibility of this happening by chance decreases dramatically, and it can be said that this was likely a real mutation.

This introduces one of the most important measures of accuracy in the next-generation sequencing pipeline. Perfect accuracy in read mapping suggests that all the reads are assigned to the actual location from which they originated; low accuracy occurs when the assignment is near-random. Without high accuracy in the mapping stage, one cannot be certain that a mutation was not identified because of a poor mapping job. Achieving commensurate gains in mapping accuracy is important for many biological problems, where single mutations (single nucleotide polymorphisms, or SNPs) can be the difference between experiment and control.

To increase the signal-to-noise ratio, DNA sequencing pipelines require a certain *coverage depth *at each base, an amount anywhere between 10 and 30x [3]. For a small organism such as bacteria or other prokaryotes, this is fairly easy; however, for an organism with a longer DNA sequence (such as the 3.2 billion bp human genome), this requires over 90 billion sequenced bases.

With the high volume of data being generated by the sequencing machines, heuristic algorithms have been used to speed up the mapping process. One of the most widely used of these heuristic algorithms is a program named *Bowtie*, which uses a suffix tree combined with the Burrows-Wheeler compression algorithm. This algorithm can be used for fast, and relatively accurate, string searches [4-6]. Many derivative algorithms have followed and researchers have studied alternative environments for the suffix tree, such as on the GPU [7,8] or Map/Reduce [9,10]. These algorithms have been primarily aimed towards greater speedups [11] but providing greater accuracy has been largely ignored.

Shorter reads from previous sequencing technologies often had problems of ambiguous mappings: either they belonged to one of many identical repeat regions, or single point mutations (whether by error or by the biology) caused them to align better to an incorrect location. As sequence lengths have increased to the thousands of base pairs seen in the current generation, the percent of repetitive sequences in the human genome drops to below 5%. In addition, the distance from each sequence to its closest neighbor increases, so individual sequencing errors or mutations have a much smaller impact.

With the read lengths increased to the point where we can be assured that single point mutations will not map to multiple locations, it becomes important to visit the accuracy question again. With this in mind we introduce the **A**PF **D**NA **M**apper (ADaM), a program that provides optimal accuracy for alignments. ADaM works by utilizing a combination of the heuristic suffix tree algorithm for easily aligned reads with an exact matching algorithm for hard to align sequences. For the exact search ADaM uses a metric space index: a general indexing technique that can be used to search any data with a properly defined distance function between points. The metric space index used by ADaM is called the Adaptive Projection Forest (APF) which creates a parallel index over a given input data set. While any exact method could be used with ADaM, the APF was used as it was found to be faster than other metric space algorithms and had a natural parallel representation.

The contributions of this paper are as follows. First, we describe the APF and compare it with several exact-matching indexes. Next, we show that the two-stage method used in ADaM can increase the accuracy from 1-5% on easily aligned sequences to over 30-40% on harder to align sequences. And finally, as there has been little work done in the area of exact matching for aligned sequences, we provide analysis on the important factors when creating metric space indexes over reads.

### Related work

With the inclusion of distance-based methods used in this work, there are three general methods used to solve the sequence alignment problem:

#### Hash-based methods

The first is a hash-based approach, used by BLAST [12], GNUMAP [13], and many others [14]. Short *k*-mers (generally *k *≪ the length of the sequence) from the reference genome are hashed in a preprocessing step, storing each *k*-mer and the location from which it came as a key-value pair. These locations are used as seeds for a full reference-read alignment, generally using a banded Smith-Waterman or Needleman-Wunsch dynamic programming algorithm to score the alignment. The major drawback with this method is that the hash-based lookup of each *k*-mer does not allow for any errors, so as the length of *k *increases, the number of allowed mismatches decreases significantly. Having very small *k*-mers allows for high sensitivity (not missing any potential matches), but the increased number of seed locations in the hash map at each *k*-mer quickly makes this method computationally intractable. To balance between speed and accuracy, these methods usually choose a value of *k *somewhere between 9 and 15.

#### Suffix-tree methods

As has already been mentioned, the second and currently fastest approach is the suffix tree, used by Bowtie [4], SOAP2 [6], and many others. This technique uses a compression algorithm known as the Burrows-Wheeler Transform to create a suffix tree: essentially a quaternary tree (one branch for each nucleotide) with "infinite" depth. Determining if a sequence exists in this tree takes exactly *l *steps, where *l *is the length of the query sequence. However, this fast lookup does not allow for differences between the read and suffix tree representation, so programs that employ this approach implement heuristics to randomly backtrack through the possible alignment space, inserting gaps or mutations into the query sequence to find positive hits. For short reads, these methods have been the fastest and least memory intensive, and have running times several orders of magnitude faster than their counterparts. However, the backtrack process exponentially increases the search time, and can become unfeasible for longer reads, especially when gaps are included.

#### Distance-based methods

Distance-based methods use metric space indexing to divide a given object index (often referred to as *points *in a *space*) in such a way that when searching for an additional *query *object, the number of distance comparisons performed is minimized. For the read mapping problem, the index can represent overlapping *k*-mers from the genome of interest, and the query object is a next-generation sequencing read. For metric spaces, there frequently is no logical "origin," so the location of a point in the space only make sense in relation to another point, often referred to as a *pivot*. We will describe two general methods used by distance-based methods here.

**Figure 1:**
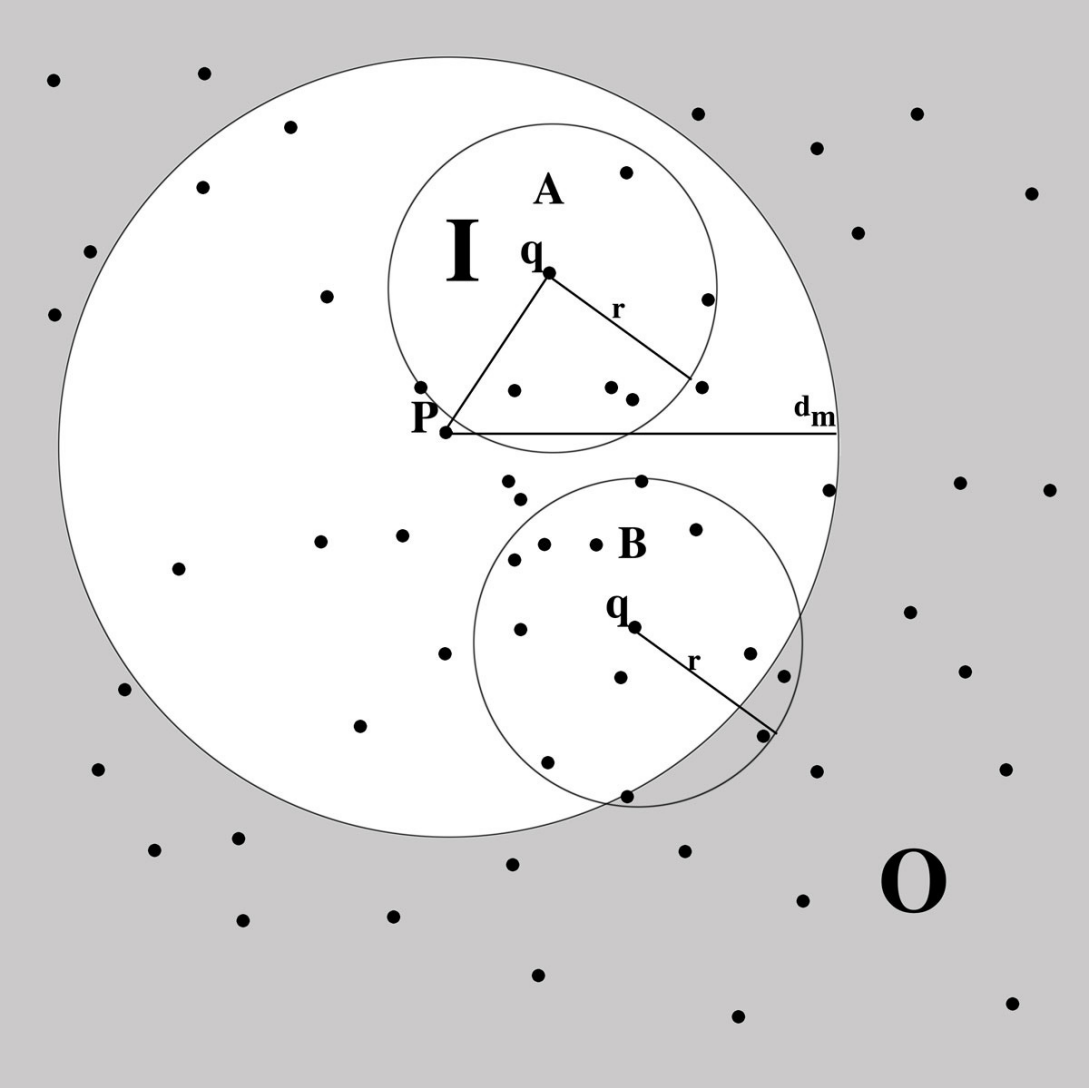
**Range search in a 2-D euclidean space**. A and B represent queries with radius *r*. I and O represent subspaces that roughly separate half the points based on their distance to the pivot point, p.

**Figure 2:**
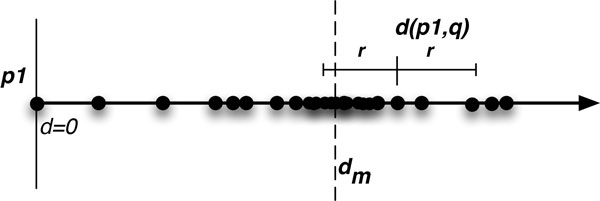
**Query point, *q*, in relation to a single pivot, *p*_1_, with query radius *r *spanning the middle distance, *d_m_***.

The *vantage-point trees *(VP Trees) work as follows. A tree is constructed with internal nodes containing pivots and external branches containing index points. During the index construction at each node, one or more pivots are selected from the data. The space is then separated into subsets as a function of their distance to the pivot, as can be seen in Figure 1. Given a query point, it is then possible, utilizing the triangle inequality, to determine whether a number of branches in the tree can be excluded. The distance from each data point to each pivot forms a mapping of the data into $R^{k}$ where *k *is the number of pivots chosen. For example, in the seminal VP Tree, each index node stores a single pivot, *p*, and the median distance, *d_m_*, and maps the data into $R^{1}$ based on the distance to *p *[15], as in Figure 2. From this, two child nodes are created, and the algorithm is then recursively applied, forming a binary tree.

The SA (Spatial Approximation) tree was designed to work for "harder" datasets where the data distribution has a histogram of distances that are extremely concentrated [16]. The tree is built by creating edges between points; the edges are chosen such that the minimum number of edges is created for all possible queries. For a given query, a random point in the SA-tree is chosen and its neighbors evaluated for the closest match. The query then moves to the closest match and the search is repeated until no other point is closer.

### Ensemble approach (ADaM)

Many areas of research have seen an ensemble perform better than any method separately (the Netflix problem [17], for example). Instead of trying to tackle the problem of referenced assembly head-on, this research employs a two-step method. First, since many sequences in a data set can be matched back to a reference genome confidently with very few mismatches, we use the suffix tree approach (specifically, Bowtie2 [4]) to align these "easy" sequences. In a normal sequencing run with low error rate, there will only be a few sequences that didn't align with high confidence, and these will be aligned with our slower but optimal algorithm, the APF, a distance-based method that provides guarantees that the results are the most accurate. With this combined approach, the overall accuracy is raised, providing base-pair accuracy even in regions of fairly low coverage.

## Methods

There are many different algorithms for string searching and pattern matching that will match sequences with various numbers of differences. However, the purpose of this program, ADaM, is to show the improvements that can be gained with an *exact *range query. Given a user-specified range, ADaM will return *all *matching sequences with zero false negatives.

Under the hood, ADaM contains a forest data structure known as the APF (Adaptive Projection Forest). The APF is based off of the Excluded Middle Vantage Point (EMVP) tree described in depth in [18-20], but has been extended to provide a greater branching factor, better control over the number of trees produced, and guaranteed bounded search times for range searches below the given radius.

### Euclidean space vs metric space

Metric-space indexes are designed to work on data that has no euclidean representation, the most common example being a 2-d graph with an origin at (0, 0). When searching for points in a euclidean space with dimension *d*, for example, the vector {0}*^d ^*can always be used as the origin, and all other points could be described by their *d*-dimensional distance from the origin.

DNA sequences do not have a natural euclidean representation, i.e. there is no defined origin that makes sense. Instead, the location of individual sequences in a given space are only defined *in reference to *other sequences. For example, the sequence *aaaa *does not have a pre-determined placement, but is considered a distance of *d*(*aaaa, aaca*) away from point *aaca*. The use of this generic distance function allows for different similarity measures in metric spaces (Hamming distance verses Needleman-Wunsch, for example), but also generalizes to point-space queries with point-space distance functions (such as the L2 norm). The distance function is what determines how "similar" two sequences are, and identifies where to search in the pre-compiled database. A distance function, *d*(*x, y*), on a metric space is defined with the following properties:

$$\begin{array}{cc} non\text{-}negativity & d\left(x,y\right)\geq 0\;\;\;\\ symmetry & d\left(x,y\right)\;=d\left(y,x\right)\;\;\\ triangle\;inequality & d\left(x,z\right)\leq d\left(x,y\right)\;+d\left(y,z\right)\\ identity\;of\;indiscernibles & d\left(x,y\right)\;=\;0\to x=y\; \end{array}$$

For DNA sequences, there are two typical distance functions: Hamming distance, and banded Needleman-Wunsch distance. The Hamming distance is simply a count of the number of characters that are different between two strings; because the chemical makeup of DNA is such that transitions (between *a *and *t *or *c *and *g*) are more likely than transversions (any other nucleotide pairings), differences may be weighted accordingly. Since DNA sequences form discrete metric spaces (and not continuous ones like the number line), this also increases the amount of total variation among nucleotides, and effectively also increases the query speed.

The second distance metric commonly used is the banded Needleman-Wunsch. This is the typical Needleman-Wunsch dynamic programming algorithm with the search space limited to a pre-determined number of gaps. The band limits the size of the dp-matrix, corresponding to a decrease in the number of calculations required.

### Guarantees of exact methods

Unlike the methods that apply heuristics to increase the speed in the string-searching process, the APF is a data structure for obtaining the guaranteed best accuracy. In other words, it can be shown that for any query searched in the forest, any and every element in the tree that lies within a user-defined distance on a given distance function will be returned as a result. Put more formally, for query *q*, result set *R*, set of index points *T*, distance function *d*(*a, b*), and distance range, *r*:

(1) $$\forall t\in T:t\in R\Leftrightarrow d\left(q,t\right)\leq r.$$

While a brute-force scan of all points would return a successful result, any metric-space algorithm seeks to reduce the overall number of distance calculations performed. Because of this, the measure of success or failure of the algorithm is not accuracy (all are guaranteed to be accurate), but in the running time of the algorithm or, since the running time is dominated by the distance function, the total number of distance comparisons performed.

In the next several sections, we will give an overview of some of the important features of the APF and how it differs from other metric indexes.

#### Implementing an exclusion area

The major difference between a *k*-d tree and the EMVP forest (as described by [19]) is that of an *exclusion area*. Figure 2 shows a single query, *q*, with radius *r*, plotted on a single dimension with index sequences plotted according to distance from *p*1. To reduce the search time, the sequences can be split in half, all those with distance less than the median distance, *d_m_*, placed on one branch of the tree, and all those greater than or equal to *d_m _*on another branch. However, because the query radius in Figure 2 crosses the *d_m_*, both branches must be included in the search. If this pattern continues at multiple levels in the tree, this can quickly degrade to a linear scan of all sequences instead of a *O*(log *n*) search.

**Figure 3:**
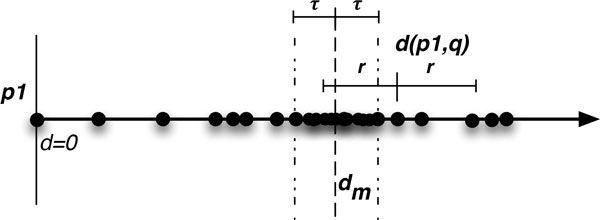
**Query point, *q*, in relation to a single pivot, *p*_1_, having exclusion radius τ** .

Adding an exclusion area of width 2 ∗ *τ *helps to mitigate this problem. As the EMVP forest is constructed, all the points *within *the exclusion area (defined by a range of ±*τ *from the median distance) will be removed, collected, and used as the starting points in a new tree. Thus, for a query with radius *r *≤ *τ*, at most one side plus a possible excluded region will need to be searched (see Figure 3).

The exclusion area is not only used to decrease the search time, but is also used to turn the tree into a forest, optimizing it for parallel processing. This forest can easily be ported to the MapReduce framework: each processor has a different tree, so individual queries are mapped once on each processor, then reduced to find the best mapping location.

#### Controlling the exclusion area

Implementing an exclusion area with a single parameter does not entirely solve the search space problem. On the one hand, fewer branches in the tree will need to be searched, but on the other, removing points within the excluded region can do more harm than good, especially considering the high frequency of points that surround the median. If more points are in the exclusion area than in the actual indexed tree, it is easy to see that this will degrade into a linear scan through the points. For example, for a random 64-bp sequence from the human X-chromosome, approximately 15% of the data lies *d_m _*± 1, and over 50% of the data lies *d_m _*± 5. If the data structure consistently excluded 50% of the data, there would be hundreds of trees and the logarithmic search time would be multiplied by a large constant. An optimal dataset for this type of build would have a bimodal distribution with only a small number of points surrounding the median, but this rarely happens in practice.

To overcome this problem, the APF uses three variables, which are set to control the maximum search radius and the number of processors being used. The variables are: the width of the exclusion area, *τ*; the maximum percentage of points in the exclusion area, *m*; and the maximum number of pivots at each node, *D*. The total exclusion region is calculated from the intersection of all pivots at that level in the tree. Each bisection of the data by a new pivot creates a new branch in the tree, for a maximum of 2*^D ^*possible tree nodes (called *children*) at the next level. At the start of the build process, either *τ *or *m *is set, and the other is calculated at runtime. If *m *was set to 0.1, a given node in the tree might have a *τ *of only 1. If *τ *was set to 2, *m *might be determined at that node to be 0.153. For data with high separability, a single pivot might be enough to confidently split the data; for highly dense data, the value of *D *might be set to 6 or 10. A major implementation detail that separates the APF apart from other indices that use exclusion is the ability to control the exclusion region and the number of points inside to create a more balanced forest.

#### Pivot selection

One of the most crucial decisions in constructing the APF is pivot selection. Since there is typically more than one pivot at each level, these pivots are ideally are selected to minimize the difference between numbers of points at each child. In other words, these pivots should be selected so that the variance *between *pivots is maximized, and the total percentage of pivots in the exclusion area is as small as possible. A poorly-selected pivot would require additional computation without providing additional information, and would result in an unbalance in the number of points at each child. For example, selecting the point {*a*}^*k *^as *p*_1 _and *c*{*a*}^*k*-1 ^as *p*_2 _provides very little information gain. Sequences that are close to *p*_1 _are also close to *p*_2_, and sequences distant from *p*_1 _are also distant from *p*_2_.

On the other hand, there is a tradeoff between build time and query time. The fastest *build *method for finding a pivot is obviously to just select a random point, but this can lead to an unbalanced tree. The fastest *query *method (optimizing tree structure) is to perform principle components analysis (PCA) with the entire set of points, as described by [21]. While this would maximize the variance between pivots, it quickly becomes computationally intractable as the size of the database increases. Further analysis of pivot selection and its impact on query time can be found later in this paper.

**Figure 4:**
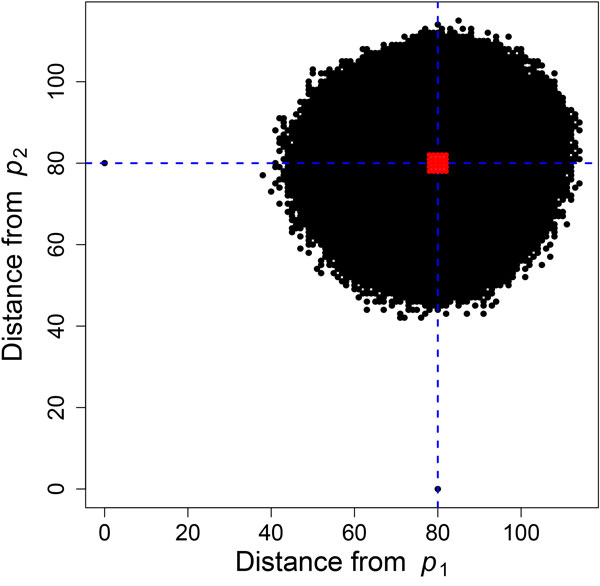
**Distances for all 100M points on human chrX, plotted as distances from pivots *p*_1 _and *p*_2_**. The median distance (*d_m _*= 80) is shown as a dashed blue line, and the exclusion points are shown as the red points in the middle (all points *d_m _*± *τ *= 2). The two outlying points at *d *= 0 are the pivot points themselves, which were selected to be at the median distance from each other.

The APF implemented in ADaM selects pivots intelligently, leveraging the data distribution. Since the data is distributed approximately normally around the median, the optimal second pivot would lie at the peak of the distribution, and would select pivots similar in a process to that done with PCA. In order to save time and avoid costly sorts and distance comparisons, successive pivots are selected in this same manner, relying on the random distribution of the data to ensure that pivots are providing information. See Figure 4 for the point distribution for two pivots, with *p*_2 _selected at the median of *p*_1_.

### Constructing and querying the APF

#### Construction

The APF is constructed by selecting a set of pivots, sorting the points according to their distance from each pivot, and using the median distance, *d_m_*, as the partitioning plane to create the exclusion area, *d_m _*± *τ *(see Figure 3). The exclusion percentage, *m*, is calculated from all the intersecting exclusion areas, and if it is too large (and the maximum number of pivots has not been reached), an additional pivot is added.

From these *d *pivots, 2*^d ^*children are created, and labeled with a number from 0. . . 2*^d ^*− 1 to identify its sector location in the *d*-dimensional space. Since the children will later become trees themselves, points are assigned to these children in the following manner: Let the label *l_i _*be the binary representation of child *c_i _*with *d *digits, each digit corresponding to a distinct pivot. If the bitwise and between 2*^j ^*and label *l_i _*is zero, then all the points assigned to this child would be *strictly less than *the median distance for pivot *p_j_*, $d_{m}\left(p_{j}\right)$. If this value is 1, the points are required to be greater than or equal to $d_{m}\left(p_{j}\right)$.

**Example 1 **If *i *= 13, *l_i _*will be the sequence 1101, and child *c_i _*would contain all points that are greater than or equal to the median for pivots *p*_3_, *p*_2_, and *p*_0_, and less than the median for pivot *p*_1_.

Points that lie in the intersecting excluded regions are saved until the tree is formed, and the process of selecting pivots and assigning points to children is recursed on each child until the size of the point set is too small, or until all points lie in an exclusion area. The exclusion points from the creation of one tree are used to form the next tree, creating a forest of AP-Trees.

#### Querying

To run a range query on the APF, the query is issued successively on each tree, returning the set of combined results. The correct traversal path through a single tree is found as follows: At each node, the distance from the query point, *q*, to each of the *d *pivots is turned into a bit vector, the reverse process of assigning points to children described above. If this is an exact query, only the single child corresponding to this bit vector need be queried, and the search space is reduced drastically. However, since a query with range *r *may not lie entirely above or below a given pivots middle distance, additional children might need to be queried. If the distance from *q *to *p _i_* plus the query range, *d*(*q, p_i_*) ± *r*, does not cross the boundary partition, it can be placed uniquely in one child node. If, however, adding the range to the query point causes the total distance to *entirely *cross the boundary (spanning 2*τ*), additional children will be searched.

**Example 2 **Let $d_{m}\left(p_{j}\right)$ be [16, 15, 14, 15], for pivots [*p*_3_, *p*_2_, *p*_1_, *p*_0_], and let *τ *= 2. Let *d*(*q, p_i_*) be [26, 18, 14, 2], for each *p_i_*. For a query of range *r *= 4, the results lie strictly below *p*_0_, both above and below *p*_1 _(because *d*(*q, p*_1_) ± *r *= [10, 18] spans $d_{m}\left(p_{1}\right)\pm \tau =\left[12,16\right]$), and strictly above *p*_2 _and *p*_3 _(because the boundary line, *d*(*q, p*_2_) − *r *= 14, is still greater than $d_{m}\left(p_{2}\right)-\tau =\;13$). The query for *q *± *r *would only need to visit two children: 0011 and 0111.

Each query is executed on each tree, and the results are aggregated and returned to ADaM. In theory, a marker for whether the queries spanned the exclusion area could be set, and subsequent trees only need be searched if this area was crossed, but since all exclusion regions from the creation of the entire tree are combined (a decision that is shown to be empirically fastest by [18]), it is highly unlikely that such an event would occur.

### Integration into ADaM

Since the APF consists of sequences with a pre-determined length, the ADaM wrapper is needed to make it useful for reads longer than the given query sequence. The ADaM algorithm can basically be thought of as a seed and extension algorithm, where the APF selects the top-scoring seeds, ADaM extends the seed alignments to find the total score, and the genomic location with the highest score is returned. Using multiple seeds will take a linearly-increasing amount of time, but will reduce the overall number of extensions needed. Currently, ADaM only supports alignments with Hamming distance, but this can easily be extended to gapped alignments with a Needleman-Wunsch or Smith-Waterman distance function.

## Results

### Metric space index comparison

**Figure 5:**
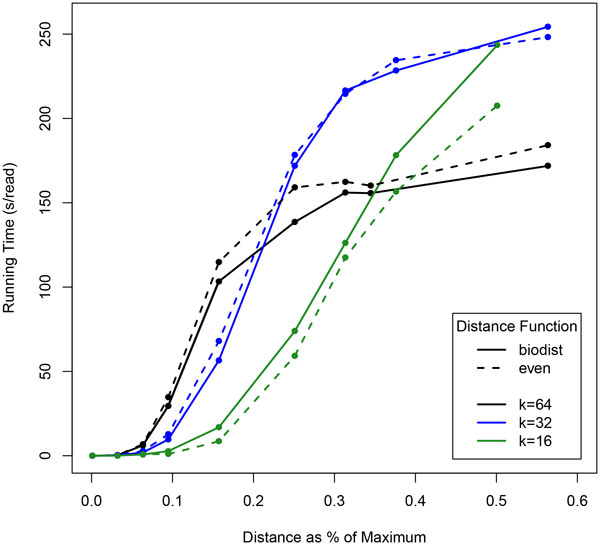
**Throughput performance for different metric-space indexes**. Performance measured as the number of distance calculations needed to find a radius that will return over the given percentage of points, so lower is better.

There are many differing generic algorithms in the field of metric-space indexing. Figure 5 shows the comparison of the APF against the MVP, EMVP, and SA trees. The comparison was done on a gaussian synthetic dataset, a DNA dataset, and an RNA dataset. The synthetic gaussian dataset contains one million vectors from ten gaussian distributions over ten dimensions. The DNA dataset consists of one million 18mers from the *Arabidopsis thaliana *genome. The RNA dataset contains one million 6mers from the *Saccharomyces cerevisiae *genome. Tests were run on 16 processors with the final result being the average number of distance calculations for the slowest processor over all queries.

Figure 5 shows that the APF index consistently performs between 5% to 25% better than the other indexes, and in some cases even more. This result shows that the APF is well suited for its use in ADaM as all of these indexes are exact and therefore speed is the most important criteria.

### Specific index with simulated data

After we determined that the APF was more efficient than other metric space indices, we built a version of the APF that was optimized for the referenced-genome mapping problem discussed in this work. In order to show the improvements over heuristic algorithms, we created a simulated dataset so we knew the correct mapping location and could thereby determine the mapping accuracy.

It is important here to note the difference between an *incorrect *and *unrecoverable *mapping error. In an incorrect mapping, the algorithm assigns the read to a location that is not globally optimal. For this kind of mapping, the correct location in the genome would give the best alignment, and thus could be recovered. An unrecoverable mapping, on the other hand, is one in which the optimal alignment (determined by Hamming or Needleman-Wunsch distance) is actually *better*. This can occur when two locations in the genome have very similar sequences, such that errors introduced in one will make it seem more similar to the other. Since the original location is no longer globally optimal, this mapping is *unrecoverable*. Obviously, it is only possible to determine which errors are incorrect with synthetic data.

As defined by the algorithm, the theoretical mapping accuracy of ADaM is guaranteed to always be 100%. However, because of unrecoverable mapping errors, the *realized *mapping accuracy might be lower. For this reason, it is not always possible to perform a "perfect" mapping, unless the reads are very long and have very high confidence. While the criteria of length and confidence have been met individually, current sequencing platforms have not been able to realize both concurrently.

In this section, we will show the improvement that comes from mapping reads with both ADaM (precise accuracy, slower speed) and Bowtie (approximate matches, very fast). In a simulated environment, it is simple to construct a pipeline where Bowtie first maps all the reads, and then ADaM maps those reads that Bowtie mapped incorrectly. In a real environment, it is impossible to tell "correct" from "incorrect" mappings, so for these experiments we use a mapping quality cutoff, $\hat{m}$, to map the set of reads, *R*, in the following procedure:

**Step 1 **Identify a mapping quality cutoff, $\hat{m}$, such that the alignment quality is poor enough to be labeled uncertain (determined from the SAM output file, a MAPQ score of 14, corresponding to a confidence threshold of roughly 95%, was used in these experiments).

**Step 2 **Align all reads, *R*, with Bowtie, using default parameters.

**Step 3 **From the Bowtie results, select all reads that either have a mapping quality less than $\hat{m}$ or were unable to be aligned by Bowtie. Let this set be *R*'.

**Step 4 **Map all reads in *R*' with ADaM, return best locations For simulated data, the accuracy of the combined ADaM/Bowtie results are tabulated.

To show the mapping accuracy of ADaM and Bowtie together, we simulated a set of 80-bp reads using Metasim [22] with a typical Illumina error profile (1x). In addition to the normal error profile, two separate error profiles were created with 2x and 10x error rates in order to determine mapping accuracy over increasingly difficult sets of reads. The results of the simulations can be found in Table 1.

**Table 1:** Accuracy of Bowtie and ADaM as the error rate changes from 1x through 10x (1x being a typical Illumina error rate, and 10x is ten times that).

	1x		2x		10x	
	**%Acc**	**RunTime**	**%Acc**	**RunTime**	**%Acc**	**RunTime**

Bowtie	95.9	11.9s	93.5	11.6s	62.2	5.48s
ADaM+Bowtie	99.6	46.7 m	99.1	56.0 m	90.6	37.0 h

Note that, even at high error, the combined approach still maintains high accuracy.

At lower error rates, Bowtie is quickly able to align all the sequences without a significant cost in accuracy (95.9% and 93.5% accuracy at 1x and 2x error rates, respectively). However, as the error rate increases to 10x, the accuracy of Bowtie drops to 62.2%. When this happens in practice, the entire data set is discarded as "unmappable." However, the ensemble approach utilized by ADaM shows that, even at high error rates, it is possible to gain high accuracy. At the 1x error rate, ADaM is able to increase Bowtie's mapping accuracy by 5%, to 99.6%, and at the 2x error rate, the combined accuracy stays at roughly 99%. So even at 1x and 2x error rates, ADaM raises the accuracy to nearly perfect accuracy.

The most notable difference is the increase in accuracy at the 10x error rate. ADaM maps a higher quantity of reads (leading to a longer running time), but is able to boost the accuracy of Bowtie by nearly 30% for a combined accuracy of 90.6%. It is important to note that, even though Bowtie's mapping results were relatively low-quality, this was not because it was impossible to achieve high accuracy for this data. The use of ADaM on these error rates brings the total accuracy up from a very low quality alignment to one that is significantly more accurate.

### Incorrect alignments

The most interesting result is the accuracy of ADaM on the reads that Bowtie maps *incorrectly*. Table 2 shows the potential accuracy for these reads, broken into categories of "skipped" and "wrong." At 1x error rate, Bowtie skipped 56 out of 10000 reads, only 1 of which was un unrecoverable mapping. At this same error rate, nearly 90% of the 346 reads it mapped incorrectly could unambiguously be mapped back to the original location. Some of the reads Bowtie mapped incorrectly appear in multiple locations in the genome. (As an aside, selecting a strategy of only reporting a random location has been shown to be just accurate as reporting a partial amount for all locations, as long as the dataset is relatively large (see, for example, [13]).) However, a large number of the reads were mapped incorrectly to a location *worse *than the originating location.

**Table 2:** Potential accuracy for reads missed and incorrectly mapped by Bowtie, as determined by the APF mapping.

	1x		2x		10x	
	**Total**	**Accuracy**	**Total**	**Accuracy**	**Total**	**Accuracy**

skipped	56	98.21	183	96.17	3427	80.84
wrong	346	89.30	463	84.88	351	62.68

At 10x, 80% of the 3,427 reads Bowtie did not map had a unique mapping in the genome. Even with a normal error profile, 55 out of 56 reads skipped by Bowtie could have been uniquely mapped. At normal error rates, 346 sequences were incorrectly mapped to a *worse *location than where they originated.

As an example, one read originated from the reverse strand of chromosome Χ at position 80216718 with only two errors. ADaM correctly mapped it back to this location as a unique match. Bowtie mapped this read to a similar location on the forward strand (chrX:62101306), but after introducing *5 mismatches*.

A second read originated from the forward strand with only three mismatches (at positions 62, 65, and 75-none in the first 61 bp), but Bowtie aligned it to a different location on the reverse strand with seven mismatches, one of which occurred at the 21st base pair. (The location in the read is important because Bowtie employs a seed-and-extend strategy, where the first *s *bases must be high quality, but the remaining bases are more flexible, following the typical Illumina error profile. For Bowtie to report a read with a worse-matching seed is therefore significant.)

This second result suggests two things. First, that the strategy employed by the suffix tree approach does not accurately capture the best possible match, even for relatively high-quality reads, and second, that there are large potential gains for exact matching algorithms.

## Discussion

In order to understand the function of the APF in the greater context of sequence mapping, we decided to specifically examine several aspects of the APF and evaluate their impact on the performance.

### Query seed length

**Figure 6:**
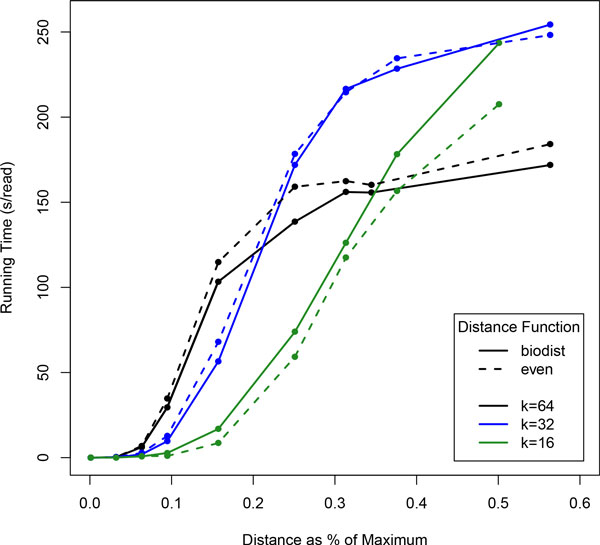
**Wall clock times with queries length *k***. Queries are of length 64 and the genome is the human Χ chromosome, about 150 Mbp. Solid lines show the *biologically relevant *distance function, and dashed lines show *even*. A distance of 10% corresponds to an alignment score of 90% possible. Queries with *k *= 32 were repeated twice along the sequence, and those with length *k *= 16 were repeated 3 times.

**Figure 7:**
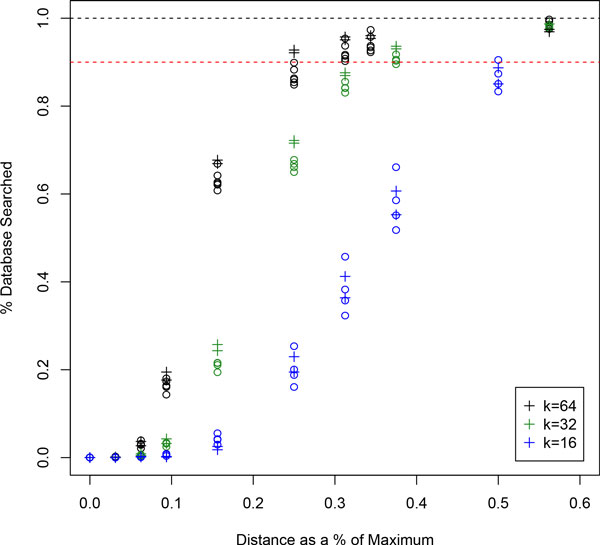
**Percentage of database searched for different runs with different values of *k***. The horizontal dotted red line shows saturation with 90% of the database searched, and the horizontal dotted black line shows a linear scan with 100%. Queries with lengths *k *= 64, 32, and 16 have reached saturation by approximately 25%, 40%, and 55%, respectively. When compared with Figure 6, the correspondence between running time and percentage database searched is obvious: once the algorithm has reached saturation level, the running times plateau.

The length of the query seed, *k*, has a large impact on query time, especially as the range of the query increases. Figure 6 shows the change in running time for several different sequence lengths as the range increases, and Figure 7 shows the percentage of database searched as the range increases. At the beginning, shorter sequences perform significantly better (at a range of *r *= 0, *k *= 64 has a running time of approximately 10 seconds, whereas *k *= 16 has a running time of only 7.5). While it is true that toward the end, longer queries do better, it can be seen when comparing with Figure 7 that the plateau in the *k *= 64 line only exists because it has reached saturation, i.e. on average, over 90% of the entire database is being searched with each query.

On the other hand, there are some potential gains for using longer sequences. For a given length *k *and the number of characters different, *r*, the size of the *expected query ball *for any given query, *q*, (the number of different sequences within *r *characters different from *q*) can be given by the following formula (adapted from [23]):

(2) $$B\left(k,r\right)=\overset{r}{\underset{i=1}{\sum }}\left(\begin{array}{c} k\\ i \end{array}\right)\;{\left(n-1\right)}^{i}$$

where *n *is the number of possible values to change (4 in this result, corresponding to the number of different nucleotides).

While *B*(*k, r*) grows faster for larger values of *k*, the *relative *number of points (*B*(*k, r*)*/k^n^*) grows *slower *for larger values of *n*. So for a database that is much smaller than *k^n^*, the estimated number of results from a given query will also be smaller for larger *n*. In ADaM, finding the best match for a sequence of length 64 will require one query with range *r *at *k *= 64 and four faster queries with *k *= 16 and *r*' = *r/*16. However, the number of seeds with *k *= 16 is much higher, so the extension process will take longer.

### Values of *τ*

The motivating advantage of the exclusion area of the APF is to limit the average number of distance comparisons for each range query. Originally, it was thought that the value of *τ *could be optimized according to the range, *r*, of the queries. For example, a range query with *r *= 0 might be best with a *τ *of zero, but a range of *r *= 12 mismatches might be best with *τ *= 12. As the size of the database grows, however, two factors prevent us from being able to find the optimal value of *τ*. First, it takes significantly longer to create an APF with a larger value of *τ*. The build time for *τ *= 0 is half a day, but the build time for 10 mismatches in database with sequences of length 64 is longer than 5 days (the hard limit for walltime on the machine we were using). The second factor that prevented us from identifying an optimal value for *τ *is that the running time is more significantly different between values of *r *than it is for values of *τ*. Thus, in order to reduce the overall running time, even the best-performing value of *τ *would take several times longer than the worst-performing value of *τ *for a smaller range.

The lack of an optimal value of *τ *also contributes to an inability to precisely bound the running time of this algorithm. Theoretically, the APF should have a complexity of $O\left(n^{\left(D-1\right)/D}\right)$ (where *D *is the number of pivots at each level), but practically the optimal value of *τ *is much smaller than the desired search radius, so the bounds are not empirically visible.

From these results, it would suggest that the smallest possible value of *τ *should be selected. However, a secondary reason for increasing the value of *τ *is to intelligently spread the data across multiple trees to better distribute the data in parallelization. An exact query on a binary tree of size *n *would take log_2 _*n*. Randomly distributing the data into *t *trees would require *t *∗ log_2 _*n/t *= *t *∗ (log_2 _*n *− log_2 _*t*). Because of this constant multiplier, *t*, it will always be more optimal to keep the data in one tree. As a general rule, the number of trees in the APF increases nearly linearly with the increase in *τ*. Because there is no significant advantage in running time for lower values of *τ*, this presents an elegant method for dividing the data into smaller MapReduce tasks without the corresponding constant multiplier.

### Pivot selection

One of the major decision criteria for building the APF is choosing the best set of pivots. Done well, there will be few pivots, and equal numbers of points in each of their children. The optimal forest will have a reasonable number of wide, bushy trees. To show the impact proper pivot selection can have on overall runtime, we used three different pivot selection techniques on a smaller genome (1 Mbp from the *C. elegans *genome). The first, *Optimal*, is the method described in this paper, of selecting a pivot point the median distance from the previous pivot. *Random *simply selects a random point, and *Poor *selects the *closest *point to the previous pivot (the opposite reasoning for *Optimal*).

**Figure 8:**
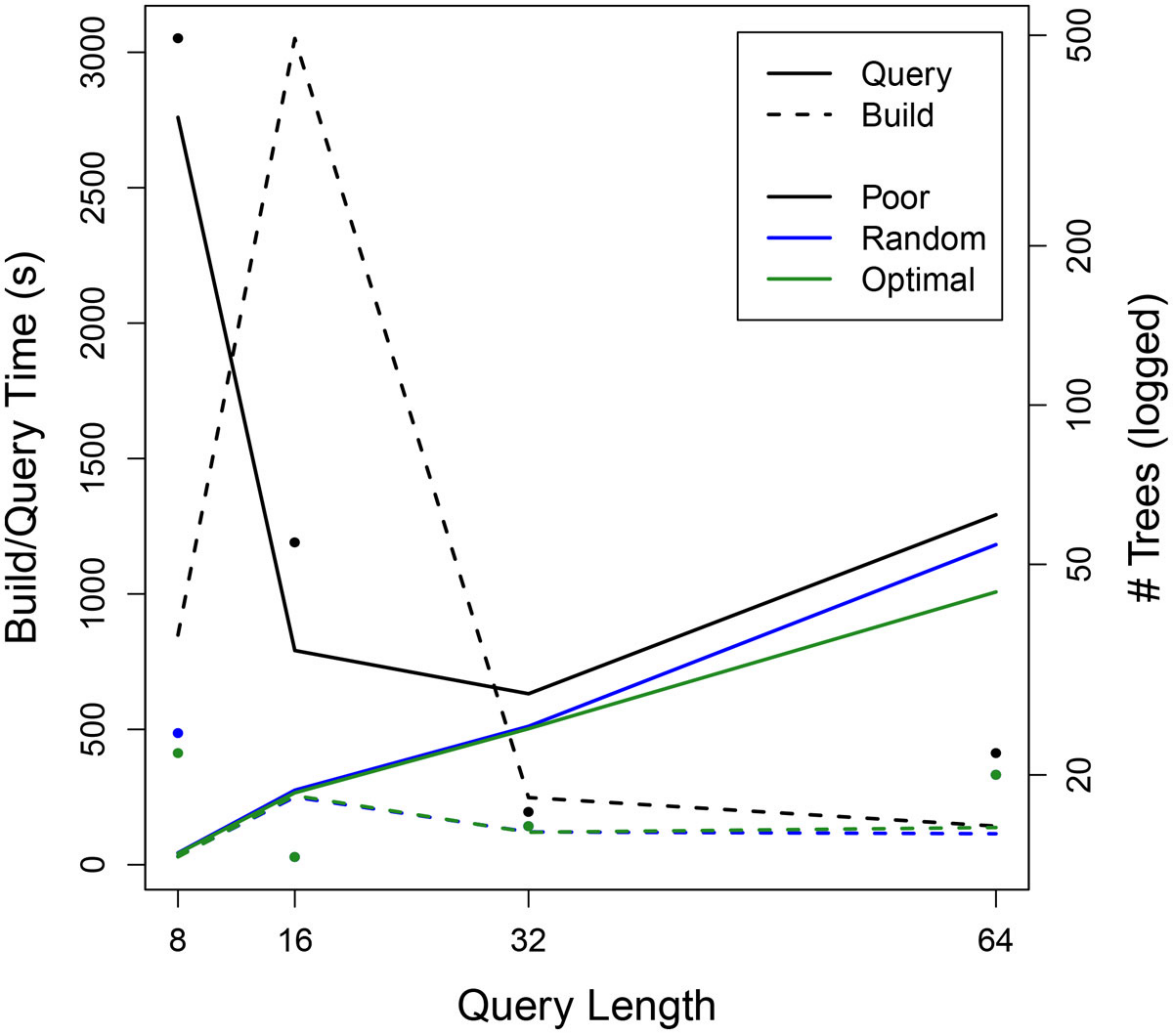
**Query and build times for different pivot selections and different lengths of query sequences**. Also included are points for the number of trees for each build. High number of trees typically corresponds with longer query times (as is the case with *Poor *selection and query length of 8, where the number of trees is 493). Random and Optimal have the same number of trees at *l *= 16, 32, and 64.

As can be seen in Figure 8, the pivot selection type has a varied impact on the build and running time. With a query length of 8 (there are only 65 k different 8nt sequences possible), the Poor selection criteria made 493 trees. While the build time was small enough to not see a noticeable difference, the query time was substantially different, as each of these trees were not full and bushy. The build time for query length 16 was even more noticeable, as the database size was much larger (915 k unique sequences). As the length of sequences increased, the selection criteria became less important, as even the closest sequences (especially in this smaller dataset) are a relatively large distance away. However, the poor pivot selection method always performs worse than the random selection method, which in turn does worse than the optimal method.

### Specifications

Tests were run on a single server with two eight-core Intel Sandy Bridge processors (2.2 GHz). The server had 32G of 1600 MHz memory and 6.14 Terabytes of storage. The ADaM implementation was written in C++. While the results were done on a single machine, ADaM already is a parallel algorithm that utilizes all available computing processors on that node. Since utilization of distributed clusters (or cloud computing) is increasingly important, especially for exact methods, work on ADaM for massively distributed clusters has already begun. This implementation can utilize the MapReduce environment, which allows algorithms to be distributed in a scalable fashion across large computing clusters.

## Conclusion

The major focus of the next-generation sequencing race has been in mapping speed. Many approximate algorithms have been developed to date that increasingly reduce the time without a significant loss in accuracy. However, very little work has been done to determine the benefits gained from an *exact *mapping approach. As was shown in this paper, even typical next-generation sequencing datasets can see improvements of up to 5%, and those with high error rates can see up to 30%.

On small datasets, where it may be difficult or impossible to extract enough DNA for high coverage, the impact of an additional 5% in accuracy can be the difference of identifying rare but important SNPs. Even on a large dataset, the extra boost in accuracy from the ADaM ensemble can increase the signal-to-noise ratio in highly-repetitive promoter regions. Going forward, it will be increasingly important to use high-accuracy mappers, especially as the length of reads increases. With longer reads also comes higher sequencing errors, and it will become even more important to have as few false negatives as possible. Using a combined ensemble will allow researchers to leverage speed and accuracy to realize even better results.

## Competing interests

The authors declare that they have no competing interests.

## Authors' contributions

LP and DM developed the algorithm; LP provided comparisons against existing algorithms; NC implemented the algorithm in C++ and provided the benchmark datasets. All authors read and approved the final manuscript.

## Declarations

The cost for the publication of the article will be funded by the authors.

This article has been published as part of *BMC Bioinformatics *Volume 15 Supplement 7, 2014: Selected articles from the 10th Annual Biotechnology and Bioinformatics Symposium (BIOT 2013). The full contents of the supplement are available online at http://www.biomedcentral.com/bmcbioinformatics/supplements/15/S7
